# Long-term results of endoclamping in patients undergoing minimally invasive mitral valve surgery where external aortic clamping cannot be used – a propensity matched analysis

**DOI:** 10.1186/s13019-020-01363-0

**Published:** 2020-10-14

**Authors:** Ayse Cetinkaya, Emad Ebraheem, Karin Bramlage, Stefan Hein, Peter Bramlage, Yeong-Hoon Choi, Markus Schönburg, Manfred Richter

**Affiliations:** 1 Department of Cardiac Surgery, Kerckhoff-Heart Center Bad Nauheim, Campus of the University Hospital Giessen, Benekestraße 2-8, 61231 Bad Nauheim, Germany; 2 Institute for Pharmacology and Preventive Medicine, Cloppenburg, Germany

**Keywords:** Mitral valve repair, Endoaortic clamping, Aortic clamping

## Abstract

**Background:**

Minimally invasive mitral valve surgery is standard of care in many centres and it is commonly associated with the need for cardiopulmonary bypass. Conventional external aortic clamping (exoclamping) is not always feasible, so endoaortic clamping (endoclamping) has evolved as a viable alternative. The aim of this study is to compare endoclamping (Intraclude*™*, Edwards Lifesciences) with exoclamping (Chitwood) during minimally invasive mitral valve procedures.

**Methods:**

This single-centre study included 822 consecutive patients undergoing minimally invasive mitral valve procedures. The endoclamp was used in 64 patients and the exoclamp in 758. Propensity-score (PS) matching was performed resulting in 63 patients per group. Outcome measures included procedural variables, length of intensive care unit (ICU) and hospital stay, major adverse cardiac and cerebrovascular events (MACCE) and repeat surgery.

**Results:**

The mean age was similar in the two group (62.2 [endoclamp] vs. 63.5 [exoclamp] years; *p* = 0.554), as were the cardiopulmonary bypass (145 vs. 156 min; *p* = 0.707) and the procedure time (203 vs. 211 min; *p* = 0.648). The X-clamp time was significantly shorter in the endoclamp group (88 vs. 99 min; *p* = 0.042). Length of ICU stay (25.0 vs. 23.0 h) and length of hospital stay (10.0 vs. 9.0 days) were slightly longer in the endoclamp group, but without statistical significance. There were nominal but no statistically significant differences between the groups in the rates of stroke, vascular complications, myocardial infarction or repeat mitral valve surgery. The conversion rate to open sternotomy approach was 2.4% without difference between groups. The estimated 7-year survival rate was similar for both groups (89.9% [endoclamp]; 84.0% [exoclamp]) with a hazard ratio of 1.291 (95% CI 0.453–3.680).

**Conclusions:**

Endoaortic clamping is an appropriate and reasonably safe alternative to the conventional Chitwood exoclamp for patients in which the exoclamp cannot be used because the ascending aorta cannot be safely mobilised.

## Background

Minimally invasive mitral valve surgery (MIMVS) is becoming the standard-of-care in suitable cases in many centres. It is commonly associated with the need for cardiopulmonary bypass, but conventional external aortic clamping (‘exoclamping’) is not always feasible or desirable. In this setting, endoaortic clamping (‘endoclamping’) has evolved into a viable alternative for providing aortic cross-clamping, antegrade cardioplegia and aortic root venting. In addition, a pressure lumen allows monitoring of the aortic root pressure.

Rates of endo- versus exoclamping in this setting range from 5% at our institution, to over 23% reported by Ius et al. [1], and up to 42% reported by Murzi et al. [2] Endoclamping has been associated with less fibrillatory arrest than exoclamping, with no prolongation of clamp time or pump time, and similar in-hospital, as well as late, outcomes [3]. On the other hand, aortic dissections with conversion to sternotomy, problems exposing the aorta and instability of the endoclamp have been reported as complications associated with the endoclamp procedure [1, 4]. Finally, costs for the endoclamp are higher than for the standard exoclamp.

Common practice at our institution is to clamp the ascending aorta directly with a flexible transthoracic clamp if the aorta can be safely dissected and clamped and to deliver antegrade cold crystalloid cardioplegia. In cases where the ascending aorta cannot be safely mobilised, we use endoclamping with antegrade cardioplegia. To date, we have successfully used the endoclamping device in a total of 64 patients undergoing MIMVS at our institution. To explore the relative merits of endoclamping in those who cannot undergo exoclamping in the MIMVS setting, we conducted an analysis of patients treated via MIMVS at our centre in which we matched those undergoing exoclamping to those undergoing endoclamping using propensity scoring.

## Materials and methods

This study was a single-centre, retrospective analysis of mitral valves surgeries performed at the Kerckhoff-Heart Center Bad Nauheim, Germany [5]. The study included patients undergoing MIMVS within the time period 2009–2015, involving either aortic endoclamping (Intraclude*™*, Edwards Lifesciences) or conventional aortic exoclamping. Interventions were performed by a group of five surgeons with no imbalance by approach. The study complied with the Declaration of Helsinki and its amendments. The study was assessed by the site’s ethical committee (University of Giessen, Germany) which stated that no approval and no patient informed consent was necessary because of its retrospective nature and the use of anonymised data.

### Patient and public involvement

Neither patients nor the public were involved in this analysis.

### Data, outcomes and definitions

For patients who had undergone a mitral valve procedure, all electronic medical records (including inpatient and outpatient notes and the results of any diagnostic testing) were reviewed. Clinical variables, including patient age, sex, comorbid diseases, prior cardiology procedures, echocardiographic procedures and other pertinent medical/surgical history, were recorded. Follow-up data concerning complications and echocardiography parameters were collected at the patient’s last follow-up hospital visit.

### Statistics

Data were analysed using descriptive statistics, with categorical variables presented as mean values with standard deviation or as frequencies (%), and continuous variables presented as the median and interquartile range (IQR).

Propensity score matching was performed. The propensity score for each patient was calculated by logistic regression with adjustment for 6 key baseline variables (hypertension, diabetes, coronary artery disease, prior myocardial infarction within 90 days, prior aortic valve implantation and emergency indication). When matching patients 1:1 a difference in propensity score of 0.01% (0.0001) was tolerated.

Comparisons between the endocamp and exoclamp groups were carried out using t-test or Mann-Whitney U test for continuous variables and a Fisher’s exact or Chi-square test for categorical variables. Survival analyses were presented as Kaplan-Meier curves. Hazard ratios (HRs) were calculated by Cox regression. In all cases, a two-tailed *p*-value of < 0.05 was considered statistically significant. All statistical tests were performed using IBM SPSS Statistics software version 24.0 (IBM Corporation, Armonk, New York, USA).

## Results

**Fig. 1:**
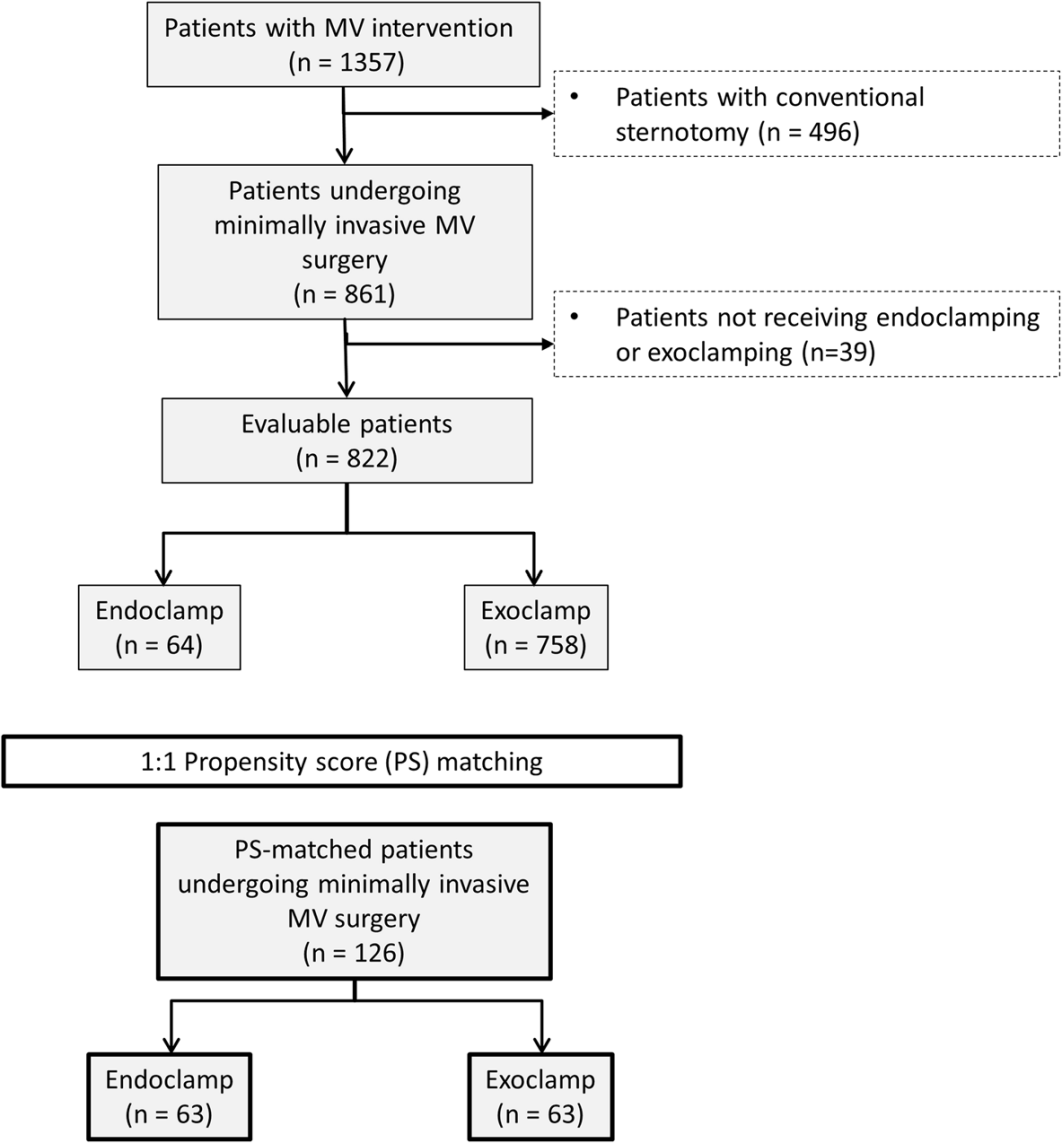
Flow Chart. MV, mitral valve; PS, propensity score

Among 822 consecutive MIMVS procedures performed at our centre between 2009 and 2015, the endoclamp was used in 64 patients and the exoclamp in 758 patients (Fig. 1). Propensity score matching resulted in 63 patients per group.

### Patient characteristics

In the overall (unmatched) population, patients had a mean age of 63 years and 42.3% were female. Patients tended to be highly symptomatic, with 68.5% being in New York Heart Association (NYHA) class III/IV (Table 1). Comorbidities were common, including atrial fibrillation (31.5%) and pulmonary hypertension (14.0%). There were a few differences between the groups in the unmatched population; however, PS-matching resulted in two similar groups of patients without any significant differences in baseline characteristics (Table 1). In the PS-matched cohort, the mean age was 62.2 years in the endoclamp group and 63.5 years in the exoclamp group (*p* = 0.554). The two groups were generally similar in terms of mitral valve pathology and other echocardiographic parameters (Table 2).

**Table 1:** Patient characteristics

	Total patient population				PS-matched patient population		
	Total ***N*** = 822	ENDOCLAMP ***N*** = 64	EXOCLAMP ***N*** = 758	***p***-value	ENDOCLAMP ***N*** = 63	EXOCLAMP N = 63	*p*-value
Age in years	63.0 ± 12.1	62.3 ± 11.4	63.0 ± 12.2	0.675	62.2 ± 11.5	63.5 ± 13.1	0.554
Female gender, %	42.3	39.1	42.6	0.581	39.7	34.9	0.581
BMI (kg/m^2^)	26.2 ± 4.5 (570)	27.2 ± 5.1 (41)	26.1 ± 4.4 (529)	0.133	27.3 ± 5.2 (40)	25.9 ± 4.7 (53)	0.179
CV risk factors							
Hypertension, %	55.7	65.6	54.9	0.097	65.1	65.1	1.000
Dyslipidemia, %	21.3	25.0	21.0	0.454	23.8	31.7	0.320
Diabetes mellitus, %	7.3	15.6	6.6	**0.020**	14.3	14.3	1.000
Comorbidities general							
Creatinine (mg/dL)	0.9 ± 0.3	0.9 ± 0.3	0.9 ± 0.3	0.977	0.9 ± 0.3	0.9 ± 0.5	0.114
Kidney failure^a^, %	0.5	0.0	0.5	1.000	0.0	3.2	0.496
Stroke, %	5.1	1.6	5.4	0.244	1.6	6.3	0.365
COPD, %	11.9	14.1	11.7	0.582	14.3	19.0	0.473
PAD, %	2.4	0.0	2.6	0.393	0.0	3.2	0.496
Comorbidity cardiac							
Atrial fibrillation, %	31.5	32.8	31.4	0.815	31.7	38.1	0.455
Coronary artery disease, %	7.7	18.8	6.7	**0.002**	17.5	19.0	0.818
Prior MI (≤ 90 days), %	0.9	3.1	0.7	0.097	3.2	1.6	1.000
Prior AVR, %	0.6	3.1	0.4	0.051	1.6	1.6	1.000
Prior CABG, %	0.7	0.0	0.8	1.000	0.0	1.6	1.000
Prior pacemaker, %	1.1	0.0	1.2	1.000	0.0	3.2	0.496
NYHA class III / IV, %	68.5	65.6	68.7	0.612	65.1	66.7	0.851
CCS class III / IV, %	2.2	0.0	2.4	0.388	0.0	6.3	0.119
Pulmonary hypertension, %	14.0	10.9	14.2	0.463	11.1	17.5	0.309
Emergency indication, %	4.5	9.4	4.1	0.061	7.9	7.9	1.000
LogEuroSCORE I, %	3.1 [1.5–6.4]	2.8 [1.5–5.5]	3.1 [1.5–6.4]	0.489	2.8 [1.5–5.5]	3.5 [1.6–9.2]	0.115

*Legend:* values are reported as percent or mean ± SD; *AVR* Aortic valve replacement, *BMI* Body mass index, *CABG* Coronary artery bypass graft, *CCS* Canadian Cardiovascular society, *COPD* Chronic obstructive pulmonary disease, *CV* Cardiovascular, *MI* Myocardial infarction, *NYHA* New York Heart Association, *PAD* Peripheral artery disease, *SD* Standard deviation; ^a^Creatinine > 2.26 mg/dL

**Table 2:** MV pathology and echocardiographic parameters

	ENDOCLAMP (N = 63)	EXOCLAMP (N = 63)	*p*-value
Echocardiographic parameters			
LVEF, %	57.8 ± 9.3	58.7 ± 10.1	0.607
LVEDD (mm)	55.2 ± 6.5	57.1 ± 12.3	0.293
LVESD (mm)	35.9 ± 6.8	35.3 ± 8.5	0.702
MV pathologies			
Degenerative, %	98.4	100	1.000
Functional, %	1.6	0	1.000
Acute endocarditis, %	6.3	1.6	0.365
Annulus dilatation, %	90.5	100	**0.028**
Annulus calcification, %	4.8	7.9	0.717
MV stenosis, %	3.2	3.2	1.000
MVI ≥ grade II, %	100	96.8	0.496

*Legend:* values are reported as percent or mean ± SD; *AML* Anterior mitral valve leaflet, *LVEDD* Left ventricular end-diastolic pressure, *LVEF* Left ventricular ejection fraction, *LVESD* Left ventricular end-systolic pressure, *MV* Mitral valve, *MVI* Mitral valve insufficiency, *SD* Standard deviation

### Procedural details and outcomes

There were several differences between the groups with respect to the procedures that were performed. Anterior mitral valve leaflet reconstruction (74.6% vs. 19.0%; *p* < 0.001) and the use of loops (95.2% vs. 63.5%; *p* < 0.001) were significantly more common in the exoclamp group than the endoclamp group, whereas posterior mitral valve leaflet reconstruction was more common in the endoclamp group (76.2% vs. 55.6%; *p* = 0.015). Among concomitant procedures, left atrial appendage closure (33.3% vs. 1.6%; *p* < 0.001) and patent foramen ovale closure (22.2% vs. 1.6%; *p* < 0.001) were more common in the exoclamp group.

The median cardiopulmonary bypass time was similar in the endoclamp and exoclamp groups (145 vs. 156 min; *p* = 0.707), as was the median total procedure time (203 vs. 211 min; *p* = 0.648); Table 3. However, the median X-clamp time was significantly shorter in the endoclamp group (88 vs. 99 min; *p* = 0.042). Median length of ICU stay (25.0 vs. 23.0 h) and length of hospital stay (10.0 vs. 9.0 days) were slightly longer for patients in the endoclamp group than in the exoclamp group, but these differences did not achieve statistical significance.

**Table 3:** Procedural details

	ENDOCLAMP (N = 63)	EXOCLAMP (N = 63)	*p*-value
Mitral valve repair			
AML reconstruction	19.0	74.6	**< 0.001**
PML reconstruction	76.2	55.6	**0.015**
Annuloplasty ring	90.5	96.8	0.273
Resection	6.3	0	0.119
Loops	63.5	95.2	**< 0.001**
Cleft Plicature	31.7	28.6	0.698
Rate of successful repair ^a^	88.9	88.9	1.000
Mitral valve replacement			
Direct	9.5	3.2	0.273
MV replacement after repair failure	1.6	7.9	0.207
Biological	7.9	6.3	1.000
Mechanical	3.2	4.8	
Concomitant procedures			
Cryoablation	38.1	38.1	1.000
LAA closure	1.6	33.3	**< 0.001**
Concomitant TVR	4.8	7.9	0.717
PFO closure	1.6	22.2	**< 0.001**
ASD closure	0	0	n.a.
Myxom	0	0	n.a.
Times			
Procedure time (min)	203.0 [180.0–259.0]	211.0 [182.0–262.0]	0.648
CPB time (min)	145.0 [127.0–189.0]	156.0 [122.0–182.0]	0.707
X-clamp time (min)	88.0 [76.0–109.0]	99.0 [80.0–124.0]	**0.042**
Length of intubation (h)	11.0 [9.0–15.0]	10.0 [8.0–13.0]	0.277
Length of ICU (h)	25.0 [21.0–76.0]	23.0 [21.0–48.]0	0.246
Length of hospital stay (d)	10.0 [8.0–13.0]	9.0 [8.0–12.0]	0.411
Conversion to sternotomy	1.6	3.2	1.000

*Legend:* values are reported as percent or median [IQR]; ^a^ Three patients were excluded as they died within 72 h after the intervention (electromechanical decoupling *n* = 1, low cardiac output and rhythm disturbances n = 1, cardiogenic shock and kidney failure n = 1); *AML* Anterior mitral valve leaflet, *ASD* Atrial septal defect, *CPB* Cardiopulmonary bypass, *ICU* Intensive care unit, *IQR* Interquartile range, *LAA* Left atrial appendage, *MV* Mitral valve, *n.a.* Not applicable, *PFO* Patent foramen ovale, *PML* Posterior mitral valve leaflet, *SD* Standard deviation, *TVR* Tricuspid valve reconstruction

The conversion rate to open sternotomy approach was 2.4% and did not differ between groups (*p* = 1.000) (Table 3).There were no significant differences in the rate of procedure-related complications between the two groups (Table 4). The most common complication in both groups was atrial fibrillation (11.1% in the endoclamp group vs. 17.5% in the exoclamp group; *p* = 0.446). There were no deaths in the immediate (72-h) post-procedural period in either group.

**Table 4:** Procedure-related complications

	ENDOCLAMP (***N*** = 63)	EXOCLAMP (N = 63)	*p*-value
	%	%	
Immediate 72 h procedural mortality	0	0	n.a.
Wound infection	0	0	n.a.
Vascular complication^a^	3.2	0	0.496
Pericardial tamponade	1.6	1.6	1.000
AV block grade III	3.2	4.8	1.000
Pneumonia	3.2	3.2	1.000
Pneumothorax	3.2	0.0	0.496
Pleural effusion	1.6	1.6	1.000
Atrial Fibrillation	11.1	17.5	0.446

*Legend:* values are reported as percent; ^a^vascular complications mean complications in the groin (vascular occlusion or lymphatic fistula); *AV* Atrioventricular, *h* Hours, *MVI* Mitral valve insufficiency, *n.a*. Not applicable

### Functional outcomes

**Fig. 2:**
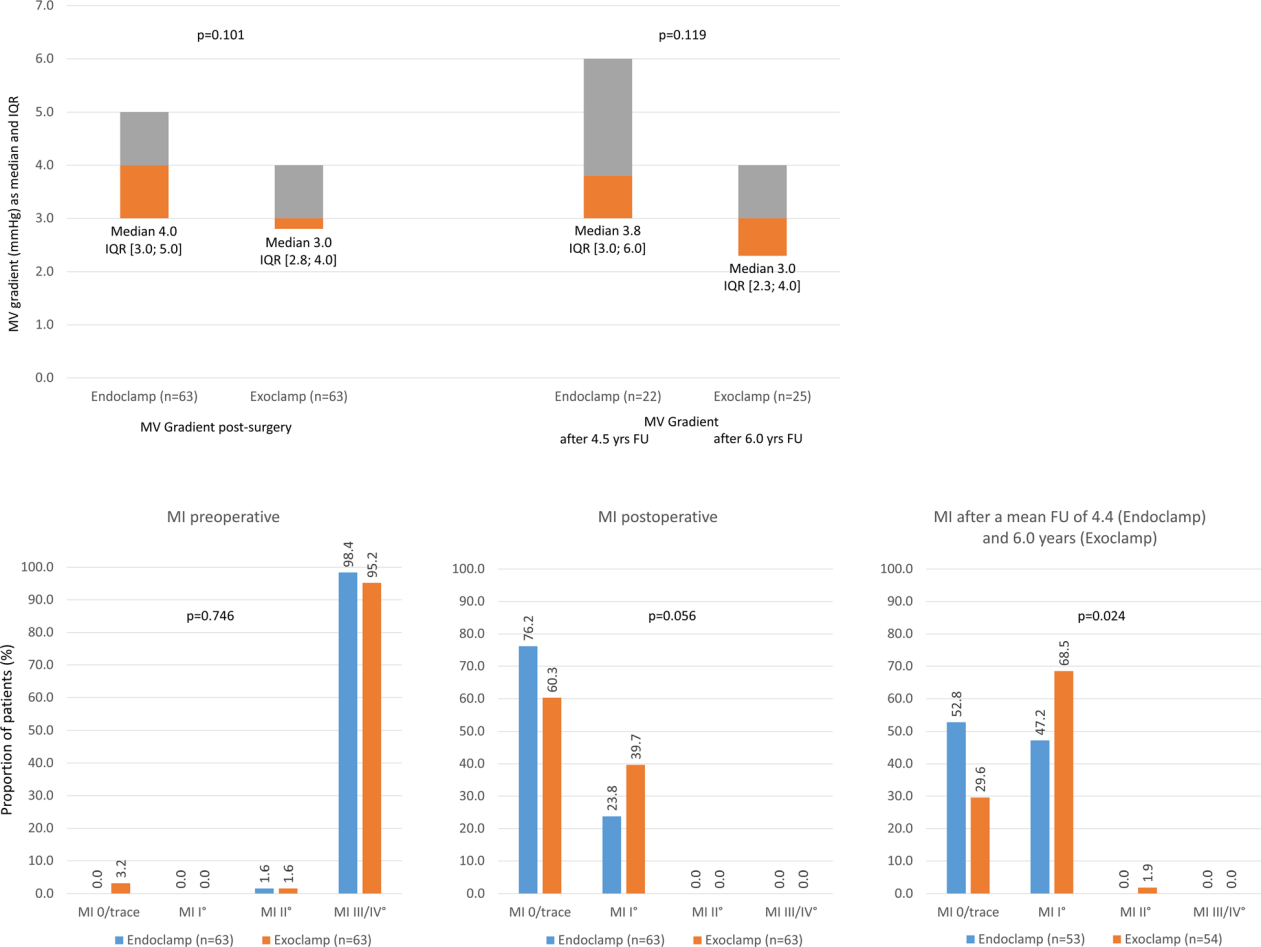
Mitral valve (MV) gradient and competency (mitral valve insufficiency; MVI). FU, follow up; IQR, interquartile range; MI, mitral insufficiency; MV, mitral valve; yrs., years

There were no significant differences in the median [IQR] mitral valve gradient between the endoclamp and exoclamp groups post-surgery (Fig. 2a).

Before surgery, there was no between-group difference in the severity of mitral insufficiency. Long-term mitral valve competency was good in both groups. However, there was trend towards a better outcome (in terms of severity of mitral insufficiency) in the endoclamp group, which achieved statistical significance during long-term follow-up (Fig. 2b).

### Clinical follow up

There were no statistically significant differences between the groups in the rates of death, stroke, vascular complications, myocardial infarction, pacemaker implantation or repeat mitral valve surgery at day 30 post-procedure (Table 5). However, event rates were low and potential differences in vascular complications and stroke may have been affected by the sample size. There were 2 deaths among the 63 patients in the endoclamp group (3.2%) compared with 4/63 (6.3%) in the exoclamp group (*p* = 0.680). Stroke occurred in 6/63 (9.5%) patients in the endoclamp group compared with 1/63 (1.6%) in the exoclamp group (*p* = 0.115).

**Table 5:** 30-day outcomes

	ENDOCLAMP (N = 63)	EXOCLAMP (N = 63)	*p*-value
	%	%	
Death	3.2	6.3	0.680
CV death	3.2	1.6	1.000
Non-CV death	0	4.8	0.244
Stroke	9.5	1.6	0.115
Acute renal failure	6.3	3.2	0.680
Myocardial infarction	3.2	1.6	1.000
Pacemaker implantation	3.2	4.8	1.000
Repeat MV surgery	0	0	n.a.

*Legend:* Values are reported as percentages; *CV* Cardiovascular, *MV* Mitral valve, *n.a*. Not applicable

**Fig. 3:**
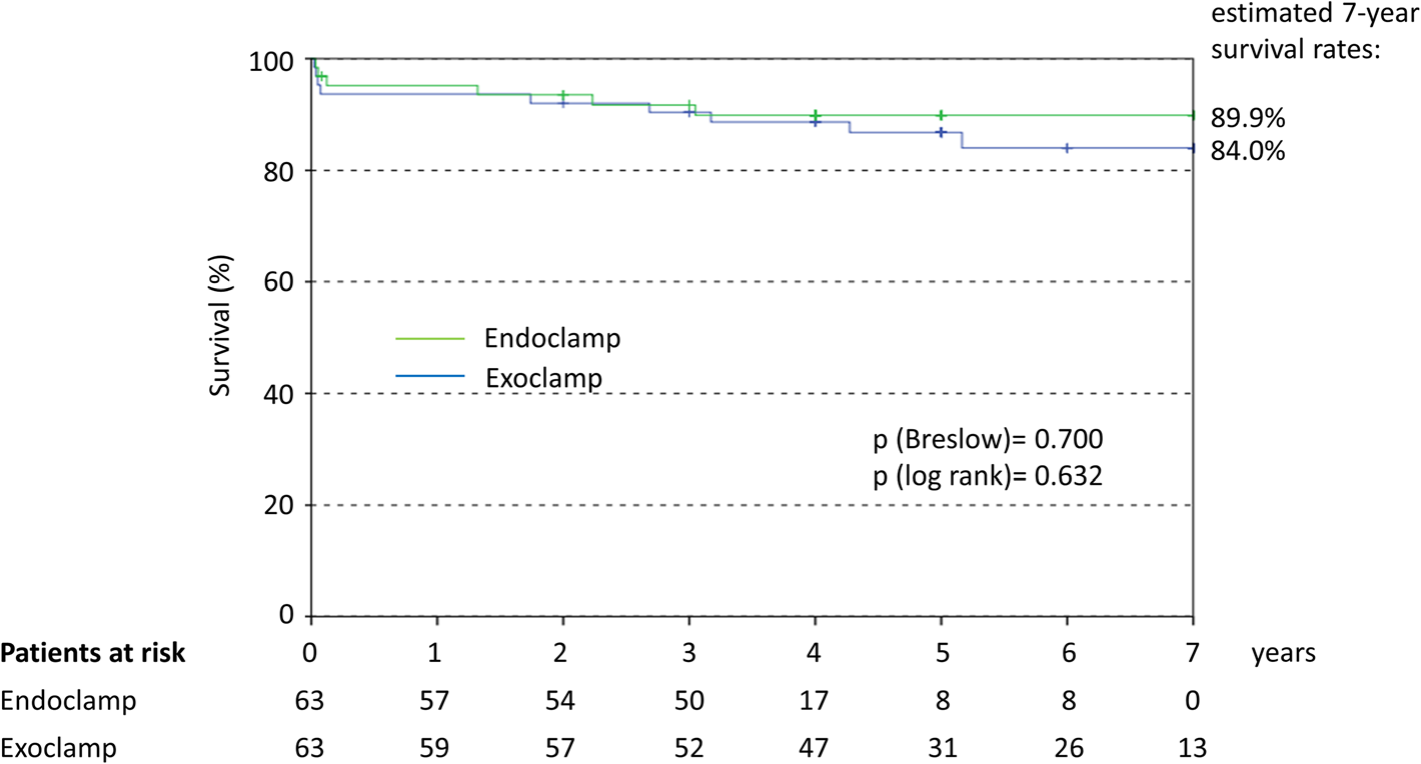
Kaplan Meier curve for long-term survival. Hazard ratio calculated by Cox regression: 1.291 (95%CI 0.453–3.680)

The estimated 7-year survival rate was similar for both groups (89.9% with endoclamp and 84.0% with exoclamp), with an HR of 1.291 (95% CI 0.453–3.680); Fig. 3.

## Discussion

Two different techniques for aortic occlusion can be used during minimally invasive cardiac surgery – external (transthoracic) aortic clamping or endoaortic balloon occlusion. Data on the use of these aortic occlusion techniques comes mainly from retrospective observational studies, and few direct comparisons have been reported [6]. We performed a PS-matched analysis of patients undergoing MIMVS at our centre to describe the outcomes of endoclamping in patients where exoclamping could not be used. Key findings included high primary MV reconstruction rates and good long-term MV competency in both groups; no significant between-group differences in complication rates or survival, which may need to be interpreted with caution because of the low absolute number with events; and shorter X-clamp and total procedure times with endoclamping.

At our institution, transthoracic clamping is more commonly performed, provided the aorta can be safely dissected and clamped; endoclamping is used in cases where the ascending aorta cannot be safely mobilised. A similar approach is used at other centres [2]. An endoclamp may also be preferred for re-operative procedures, where scarring around the aortic root can make it difficult to apply an external clamp [7–9]. There is a learning curve for the technique associated with using an endoaortic balloon, with outcomes generally improving as the number of completed procedures increases [10]. However, with good patient selection, a highly competent team, use of an appropriate insertion technique and imaging, and careful monitoring, the risk of adverse outcomes can be minimised [8, 11].

We compared outcomes after endoclamping and exoclamping using PS-matched groups to take account of baseline characteristics. We found some differences between the PS-matched groups in terms of the specific procedures that were performed: anterior mitral valve leaflet reconstruction, left atrial appendage closure and patent foramen ovale closure were all more common in the exoclamp group, whereas posterior mitral valve leaflet reconstruction was more common in the endoclamp group. The reasons for this difference are not clear, but it is possible that surgeons did not consider the endoclamp procedure to be appropriate in some patients undergoing more complex procedures. Other studies have not reported differences in the type of operation performed between endoclamp and exoclamp groups [12, 13].

Our analysis found no significant difference in the overall procedure time or in cardiopulmonary bypass time between the two groups, but the median X-clamp time was shorter (by 11 min) in the endoclamp group. Some previous studies have found no significant difference in cardiopulmonary bypass and X-clamp times between exoclamping and endoclamping groups [12–15], whereas others have reported longer operating, cardiopulmonary bypass and X-clamp times with endoclamping [7, 16, 17]. Meta-analyses have generally found no significant difference in X-clamp or cardiopulmonary bypass times between endoclamping and exoclamping [6, 18, 19]. However, a subgroup analysis found some differences depending on the cannulation used for endoclamping: X-clamp and cardiopulmonary bypass times were shorter with exoclamping compared with endoclamping with femoral cannulation; however, X-clamp time was shorter with endoclamping with aortic cannulation compared with exoclamping, and cardiopulmonary bypass time did not differ between exoclamping and endoclamping with aortic cannulation [19].

In our study, ICU and hospital stays were slightly longer in the endoclamp group than the exoclamp group (by 2 and 1 days, respectively), but the differences were not statistically significant. This is consistent with most previous studies which have found no significant difference in the length of ICU stay [13–15] or hospital stay [6, 7, 14, 15, 17], although one study reported a significantly longer hospital stay (by 2 days) after endoclamping [16] and another a significantly longer hospital stay (by 1 day) after exoclamping [13].

Successful primary mitral valve reconstruction rates were high and long-term mitral valve competency was good in both groups in our study. However, there was trend towards a better outcome with respect to valve competency in the endoclamp group, which achieved statistical significance during long-term follow-up. This is a somewhat surprising finding. Other studies have found no significant difference in the severity of mitral insufficiency between endoclamp and exoclamp groups at the time of discharge from hospital [7, 13, 15], but comparative data for this parameter after long-term follow-up are scarce.

We found no significant difference in the rates of peri-procedural complications or adverse outcomes at 30 days post-procedure between the two groups. However, 30-day event rates were low, which precludes meaningful *p*-values for the comparison at this timepoint. The rate of all-cause mortality at 30 days was numerically slightly higher in the exoclamp group (4/63; 6.3%) than in the endoclamp group (2/63; 3.2%). Previous studies and meta-analyses have not found a significant difference in 30-day all-cause mortality rates between endoclamping and exoclamping groups [6–8, 12, 13, 18–20].

Strokes occurred in numerically more patients in the endoclamp group (6/63; 9.5%) than in the exoclamp group (1/63; 1.6%) in our study, but this was not statistically significant. While further investigation is needed to better understand why the occurrence of stroke was higher in patients in the endoclamp group in this analysis, previously published reports have suggested a possible increased risk of neurological complications with the use of endoaortic occlusion compared with external clamping [12, 21]. Conversely, other studies have not reported this finding [8]. Recent meta-analyses found no significant difference in the risk of cerebrovascular accidents between the two approaches [18, 19]. Potential reasons for an increased stroke risk with endoclamping include the risk of emboli mobilisation by the guidewire, occlusion of arteries by balloon catheters that have migrated, and re-positioning of balloons without partial deflation [18]. One meta-analysis found that the relative risk of neurological events with endoclamping versus exoclamping was lower in more recent publications than in older reports, and the authors suggested this may be due to the availability of improved devices and cannulas, greater surgeon experience and improved echocardiography guidance [18]. Despite this, the long-term (7-year) survival rates for patients were similar in both groups at our centre.

Endoaortic balloon occlusion is a versatile tool with applicability for a range of cardiac surgeries [22]. In general, certain measures are vital to ensure the safe performance of endoclamping. Viability should be confirmed using preoperative imaging [22]. Transoesophageal echocardiographic monitoring is vital to control guidewire advancement and ensure correct positioning of the endoclamp [4, 10, 22]. Changing the balloon position after the initial endoclamping has been undertaken is possible under echocardiographic surveillance and with partial deflation of the balloon to avoid damaging the aortic wall [22]. It has been shown that MIMVS using endoclamping is a safe procedure even during the initial learning curve [11]. Our study confirms the feasibility and safety of endoclamping during MIMVS.

Some authors have suggested that endoclamping might be associated with higher costs than exoclamping at their centres, due to device costs, the technical complexity and a longer operative time [14, 15], whereas others have found that the endoclamping approach may be more economic [1]. However, no studies have reported specific cost data comparing the different approaches.

### Limitations

The main limitation of this observational study is its retrospective nature, making it subject to potential selection bias. Propensity-score matching was used to mitigate this but did not eliminate it completely. For example, surgeon expertise, and the date at which surgery was performed during the 6-year study period, were not taken into account. The aim of the study was to report on the outcomes of patients receiving endoclamping during minimally invasive mitral surgery, but the use of propensity-score matching matched these patients to a control of patients receiving exoclamping to put the results into perspective. As a result, the data has become a comparison of endo- versus exoclamping rather than focusing on the outcomes of endoclamping alone. The relatively small number of patients in the endoclamp cohort meant that the study had a small overall sample size after propensity matching, and therefore lacked power to fully address some issues, such as whether there was a difference in the incidence of stroke. The final limitation of this study is that it was a single-centre study, which limits the generalisability of the results.

Additional studies, including randomized controlled trials and prospective observational studies with a larger sample size and adequate power are needed to address some issues further, such as the incidence of stroke with the endoclamp procedure. Such studies may also help to further define the subgroup of patients for whom endoaortic clamping is appropriate, and clarify the technical aspects of the procedure that are key for optimising patient outcomes.

## Conclusions

Analysis of data from our centre indicates that endoaortic clamping (Intraclude*™*, Edwards Lifesciences) is an appropriate and reasonably safe alternative to the conventional Chitwood exoclamp for patients in which the exoclamp cannot be used because the ascending aorta cannot be safely mobilised.

## Data Availability

Data are available from the corresponding author upon reasonable request.
